# The Story of the Middlesex Hospital

**Published:** 1898-08-20

**Authors:** 


### THE STORY OF THE MIDDLESEX HOSPITAL.

The *Middlesex Hospital Journal* for July is mainly devoted to "The Story of the Middlesex Hospital," by Dr. Sydney Coupland, the history of which institution is carried from its foundation in 1745 to 1835, the year in which it became a medical school. Apart from the interest which every one connected with the hospital will feel in Dr. Coupland's article, the story of the hospital will appeal to the general reader as a history of the progress of hospital work from practically the commencement of the movement which has filled both London and the provinces with our modern institutions for the treatment of the sick.

The story gives us strange glimpses of hospital management and interior economy in the last century, and throws sidelights on petty jealousies existing at that time which are often amusing. The Middlesex Infirmary, as it was at first called, was started in 1745 by voluntary contributions. Its first home was in Windmill Street, in two houses rented at £30 a year. The following year found its name changed to the Middlesex Hospital. At that time the number of beds available was only 15. The next few years saw an attempt made to make the institution a lying-in hospital, terminating for the time being in one-third of the beds being set aside for that purpose. In this early stage of the existence of the hospital the lay governors appear to have been somewhat jealous of their authority, and the archives of the institution show a resolution "That no physician, man midwife, or surgeon, or other officer or servant of this charity who shall hereafter be elected shall act as governor of this charity during his continuance in office." The edict of the then governors, such as "Ordered, that the secretary do acquaint the physicians and surgeons of an amputation on Thursday next," and "That no surgeon of this hospital do presume to perform any capital operation until after a consultation had of the physicians and surgeons of this hospital and approved of, but in cases of necessity only," and a rebuke to a surgeon who performed such an operation without consultation, read somewhat curiously nowadays—almost as curiously as the quaint titles of "Man midwife in ordinary," and "Man midwife extraordinary." "1755.—I saw the foundation stone of the present hospital building laid in the Marylebone Fields, at that time a singularly unsuitable place according to our modern notions of sanitation, close to marshy land and ponds fed by the springs from Hampstead, and in the centre of fields and waste lands used for brick making and receptacles for the refuse and rubbish of the town—to say nothing of cesspits. The Middlesex seems to have flourished in spite of its unsuitable surroundings. Although the number of cases of ague treated in it in its earlier years was large, it was not until 1757 that the patients were transferred to the new building. It is curious how little the additions and changes of intervening years have altered the aspect of the building; the general appearance of the block in 1898 is seemingly much the same as the original designs of 1755, though the east and west wings were not added till some years later. The number of beds when the new hospital was opened was sixty-four. It is noticeable that the Middlesex was the first hospital where accommodation was provided for lying-in, although shortly after its establishment came the foundation of other lying-in hospitals in the metropolis. Some years later the system of attending cases of this description at their own homes was introduced, and the number of beds allotted to them in the hospital was reduced to two, and in 1807 midwifery in-patients were finally done away with. The following years show the hospital at times in straits for funds, and it was seldom that all the wards could be kept open, and, indeed, they were not all filled till 1829. The management were at times reduced to various forms of retrenchment. Leeches, however, were apparently looked on as necessities, for as late as 1821 the number of leeches expended during the year amounted to 36,100—nearly one hundred a day—and the committee recommended that "the apothecary should be desired to see that the leeches after being used are properly preserved for a second application." In 1789 the uncleanliness of the hospital called forth severe strictures from John Howard, which were bitterly resented by the governors. They flatly contradicted Mr. Howard's assertions, but at the same time set to work to thoroughly cleanse and repair the whole hospital, so presumably the charges were not foundationless.

In 1791 the hospital received £1,000 from the proceeds of the Westminster Abbey festival, and in recognition of this the board gave the name of "Handel" to one of the wards, a name which was preserved as the title of one or other of the wards for nearly 100 years. The scarcity of money and the dearness of food due to the Napoleonic wars affected the hospital, as was to be expected, and the year 1800 finds the hospital staff with their allowance of bread and butter reduced. This reduction was also made to apply to the sick French immigrants, to whom two wards of the hospital were at that time allotted. The history of the cancer ward opened in 1792 is interesting. This ward seems to have offered a field for experiment most tempting to investigators, and regulations had to be issued restricting experimental treatment. This cancer ward was the object of a severe attack by the *Lancet* some years later. Space does not permit of any reference to the history of the teaching school, now one of the foremost in the kingdom. The conclusion of the article in a subsequent number will be awaited with interest.

